# Integrative PheWAS analysis in risk categorization of major depressive disorder and identifying their associations with genetic variants using a latent topic model approach

**DOI:** 10.1038/s41398-022-02015-8

**Published:** 2022-06-08

**Authors:** Xiangfei Meng, Michelle Wang, Kieran J. O’Donnell, Jean Caron, Michael J. Meaney, Yue Li

**Affiliations:** 1 Department of Psychiatry, Faculty of Medicine and Health Sciences, McGill University, Montréal, QC Canada; 2 Douglas Research Centre, Montréal, QC Canada; 3 Yale Child Study Center & Department of Obstetrics Gynecology & Reproductive Sciences, Yale School of Medicine, Yale University, New Haven, CT USA; 4 Child & Brain Development Program, CIFAR, Toronto, ON Canada; 5 School of Computer Science, McGill University, Montréal, QC Canada

**Keywords:** Depression, Clinical genetics

## Abstract

Major depressive disorder (MDD) is the most prevalent mental disorder that constitutes a major public health problem. A tool for predicting the risk of MDD could assist with the early identification of MDD patients and targeted interventions to reduce the risk. We aimed to derive a risk prediction tool that can categorize the risk of MDD as well as discover biologically meaningful genetic variants. Data analyzed were from the fourth and fifth data collections of a longitudinal community-based cohort from Southwest Montreal, Canada, between 2015 and 2018. To account for high dimensional features, we adopted a latent topic model approach to infer a set of topical distributions over those studied predictors that characterize the underlying meta-phenotypes of the MDD cohort. MDD probability derived from 30 MDD meta-phenotypes demonstrated superior prediction accuracy to differentiate MDD cases and controls. Six latent MDD meta-phenotypes we inferred via a latent topic model were highly interpretable. We then explored potential genetic variants that were statistically associated with these MDD meta-phenotypes. The genetic heritability of MDD meta-phenotypes was 0.126 (SE = 0.316), compared to 0.000001 (SE = 0.297) for MDD diagnosis defined by the structured interviews. We discovered a list of significant MDD - related genes and pathways that were missed by MDD diagnosis. Our risk prediction model confers not only accurate MDD risk categorization but also meaningful associations with genetic predispositions that are linked to MDD subtypes. Our findings shed light on future research focusing on these identified genes and pathways for MDD subtypes.

## Introduction

The complex nature of major depressive disorder (MDD) significantly hinders its risk categorization [1, 2]. Although many risk prediction algorithms for MDD have been proposed [3–7], their prediction accuracy cannot meet the need for accurate identification and classification of MDD. The advancement in machine learning provides opportunities to address issues that cannot be handled by conventional statistical approaches [8]. However, most of these prediction models rely on a limited number of pre-determined self-reported psychological and social factors, which can be subjective and selective. This might result in an incomplete understanding of MDD. It is critical to apply a holistic approach to consider all the possible combinations of these attributable predictors in characterizing risk. However, this approach can lead to a combinatorial feature space that grows exponentially to the number of predictors, which easily becomes intractable when dealing with a few hundred features. Notably, many predictors share common information and can be potentially categorized under much fewer latent dimensions. It is reasonable to postulate that MDD patients with similar clinical manifestations could also be grouped in some latent memberships. Unsupervised learning is a class of dimensionality reduction techniques, which can be used to generate representations of the underlying structure of the data and are often used to obtain insight into the underlying structure of complex data [9]. Blei et al. proposed a topic modeling approach, which can produce highly interpretable latent dimensions in the form of latent topics for the ease of downstream analysis [10].

In this present study, we aim to develop MDD risk prediction by comprehensively identifying meaningful predictors from a wide range of clinical, biological, and psychosocial attributes. We also conduct genome-wide association studies (GWAS) over the MDD phenotype defined by the risk prediction algorithm and compare the power of this approach with the traditional GWAS approach using only the binary MDD phenotype and genotype information.

## Materials/subjects and methods

This study followed the Transparent Reporting of a Multivariable Prediction Model for Individual Prognosis or Diagnosis (TRIPOD) reporting guideline for reporting multivariable prediction model development and validation [11]. Table S1 provides a list of abbreviations and their explanations.

### Study cohort

Data analyzed were from the Zone d'Épidémiologie Psychiatrique du Sud-Ouest de Montréal (ZEPSOM) cohort, which is a large-scale, longitudinal, community-based, population cohort from the Southwest Montreal [12]. Methods for recruitment, interview, and measurement have been mentioned elsewhere [12–14]. The present study utilized data from its fourth and fifth data collections, which had complete information on major depression and genetic information. ZEPSOM was approved by the Research Ethics Board of the Douglas Mental Health University Institute (#IUSMD- 06-33; #IUSMD-16-64). All participants gave informed consent. The age of the study cohort ranged from 18 to 78 years (mean = 50, standard deviation = 14). The majority of the present study cohort were females (*n* = 850, 63%), French-Caucasian (*n* = 832, 62%), and were not diagnosed with MDD (*n* = 1 035, 77%). Compared to participants without MDD, females, without university degrees or being French-/English-Caucasian were more likely to have MDD.

### Measures

#### Clinical, psychological, and social predictors

A series of validated questionnaires were used to measure clinical, psychological, and social predictors. The present study used a total of 515 predictors, including psychological distress, family history of mental disorders, use of psychotropic drugs in the past 12 months prior to the interview, lifetime stressful events, early life adversities, social support, impulsivity, coping, life satisfaction, aggressivity, chronic physical diseases, sociodemographic characteristics, spirituality, cognitive impairment, and perceived need for mental health care. Responses to questions were either binary (i.e., “yes” or “no”), categorical (e.g., Likert-type scale), or continuous. Table S2 provides a full list of predictors in the present study.

#### MDD phenotype

MDD was measured by a modified version of the World Health Organization version of Composite International Diagnostic Interview (WHO-CIDI) [15], which used the Diagnostic and Statistical Manual of Mental Disorders, 4th edition (DSM-IV) and the International Statistical Classification of Diseases and Related Health Problems, 10th revision (ICD-10) definitions [16, 17]. We hereafter use “MDD diagnosis” to refer to the diagnosis based on the measurement from the WHO-CIDI.

#### Genetic variants

The genetic dataset consisted of single-nucleotide polymorphism (SNP) data from genetic sequencing. ZEPSOM was genotyped using genome-wide platforms (Global Screening Array V2, Illumina, CA, USA) according to the manufacturer’s guidelines with 200 ng of genomic DNA derived from buccal epithelial cells and our quality control procedures.

### Statistical analysis

#### Phenotypic modeling using a topic model

Continuous variables were dichotomized following a proportional split method consisting of computing a threshold based on the skewness of the data [18]. Fifteen of the continuous variables were removed because they were too skewed and could not be dichotomized properly, even after applying a log transform. Categorical variables were transformed into binary variables.

**Fig. 1:**
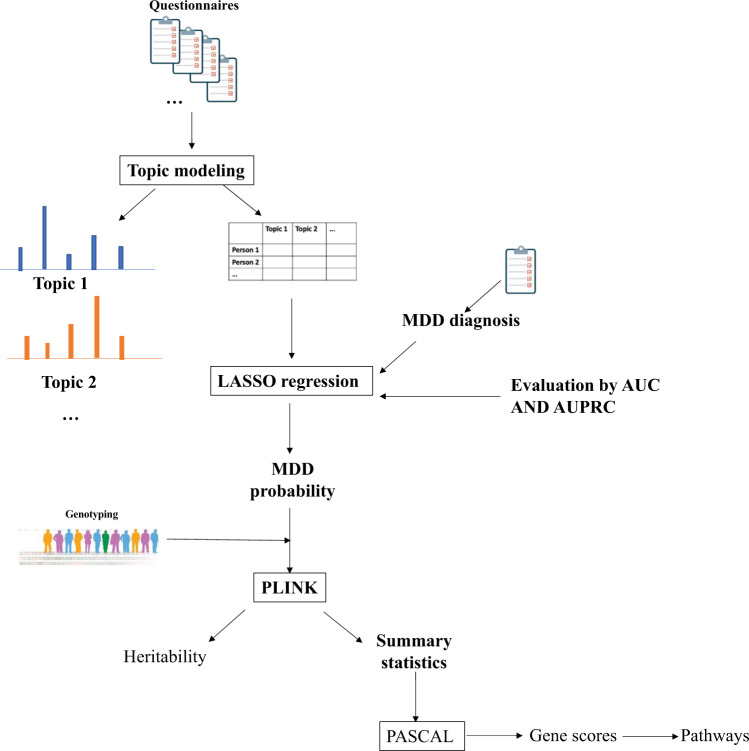
Schematic pipeline of the preprocessing and statistical analysis performed in this study.

To infer latent topics (we called meta-phenotypes) from the questionnaire data, we used the MixEHR software, which is an implementation of the collapsed variational Bayesian inference of the Latent Dirichlet Allocation (LDA) model [19]. MixEHR computes a membership matrix where each input phenotype is assigned a probability of belonging to a meta-phenotype. MixEHR was first trained on all the data except for MDD diagnosis. Then the inferred patient topic mixture (meta-phenotype membership values for each patient) was used as input to a least absolute shrinkage and selection operator (LASSO) logistic regression model, as implemented in the Scikit-Learn Python library [20]. The target variable for LASSO regression was MDD diagnosis. We experimented with the different numbers of the meta-phenotypes and found that 30 meta-phenotypes yielded the highest classification accuracy in terms of the area under the receiver operating characteristic (AUC) curve and the area under the precision-recall curve (AUPRC). We developed a phenotype- MDD probability - using the fitted probability values from the LASSO regression model regressed on the 30 meta-phenotypes. MDD probability was then used in our subsequent genetic analyses and was compared to MDD diagnosis for the GWAS power. Figure 1 shows a schematic diagram of the pipeline as a summary.

#### Statistical genetic analysis

The genotype data were preprocessed using PLINK [21]. Respondents with a low genotyping rate (<98%) and SNPs showing significant deviation from Hardy-Weinberg equilibrium (*P*-value < 1 × 10^–5^), a low minor allele frequency (MAF < 10%), or high rates of missing data (>5%) were excluded. We only used SNP data from respondents who were Caucasian. The final genetic dataset consisted of 745,201 SNPs from a total of 1083 Caucasian participants.

SNP-heritability and genetic correlation values were estimated using the GCTA software tool [22], which uses a restricted maximum likelihood algorithm (REML) [23, 24]. Summary statistics were generated with PLINK. Logistic and linear regressions were used for binary and continuous outcomes respectively. Gene scores were computed with the Pascal tool [25], and the DisGeNET database [26] was used to identify genes that have been associated with MDD in previous work.

Pascal gene scores were used to compute pairwise gene score correlations. For a pair of phenotypes, the gene scores of MDD genes were correlated with the scores of the same genes for the other phenotype. Enrichment analysis for the top (*p* ≤ 0.1) MDD genes for a meta-phenotype was done using the DAVID functional annotation tool [27, 28]. The databases used for the enrichment analysis were the Genetic Association Database [29] and the Gene Ontology Biological Processes database [30, 31].

## Results

### Inferring meaningful MDD meta-phenotypes

**Fig. 2:**
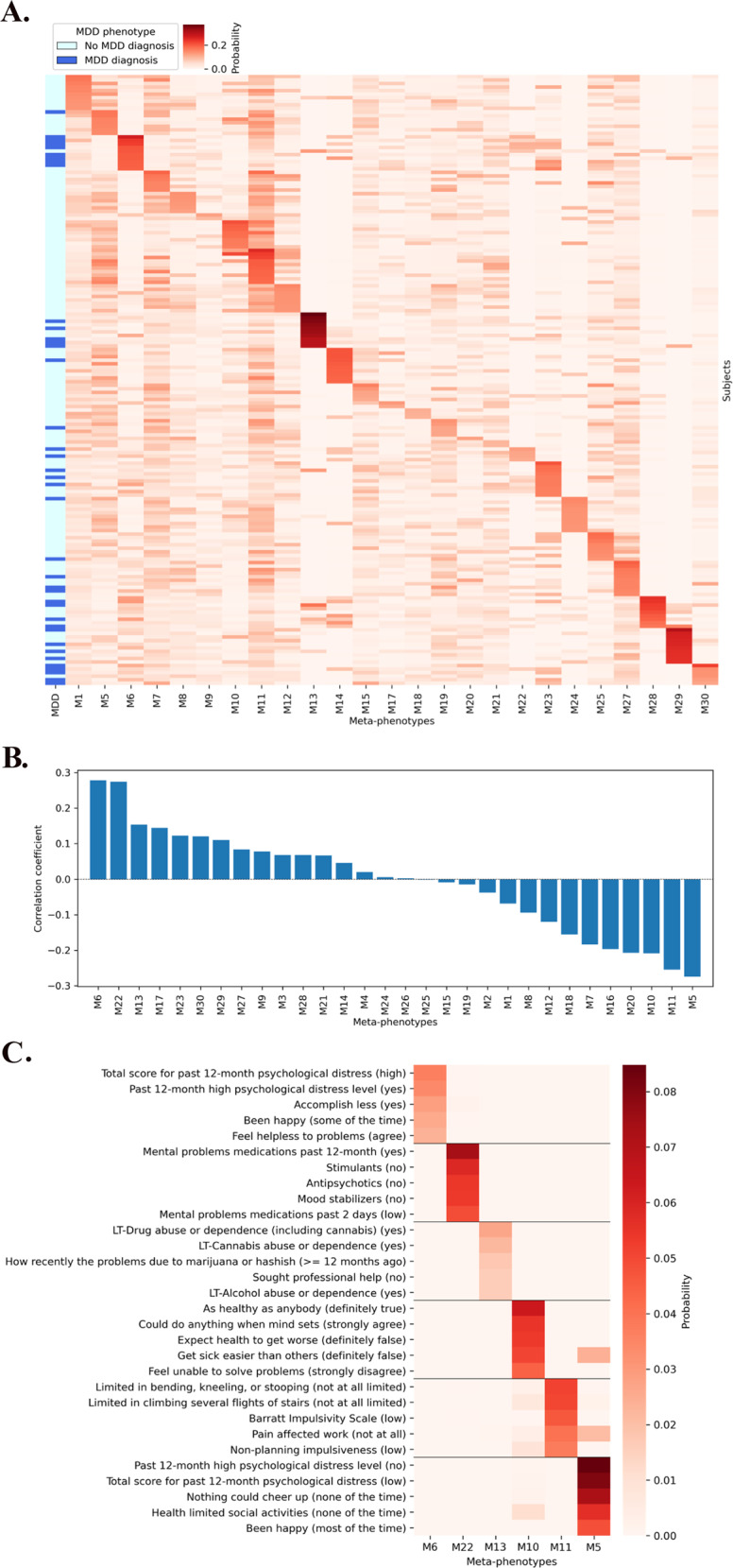
Inferring meaningful MDD meta-phenotypes. **A** Inferring 30-topic mixture of the study cohort; (**B**) Linear correlation coefficients of the 30 meta-phenotypes; (**C**) Top MDD meta-phenotype features.

We applied the MixEHR model to infer latent MDD meta-phenotypes and developed a probabilistic MDD risk prediction – MDD probability. Figure 2A provides side-by-side comparisons between the probabilities of inferred 30 MDD meta-phenotypes and MDD diagnosis based on the structured interview among a subset of respondents in the study cohort (top 10 subjects with the highest probability within each meta-phenotype). Respondents with MDD were clustered in different groups, suggesting that the latent meta-phenotypes learned by the MixEHR model discriminate MDD subtypes. We quantified correlations between the 30 MixEHR meta-phenotypes and MDD diagnosis in Fig. 2B. We focused our analysis on six meta-phenotypes that exhibit the strongest correlations (three positive ones: M6, M22, M13, and three negative ones: M10, M11, M5) with MDD diagnosis. Figure 2C displays the top five features in these most correlated meta-phenotypes. Meta-phenotypes with the strongest positive correlations were related to high psychological distress (M6), pre-existing substance abuse problems (M13), and use of medications for mental problems (M22). Meta-phenotypes with the strongest negative correlations were related to better self-perceived health (M10), limited restrictions on mobility and no/limited impulsiveness (M11), and no/low psychological distress (M5).

### Classification of MDD using inferred meta-phenotypes

**Fig. 3:**
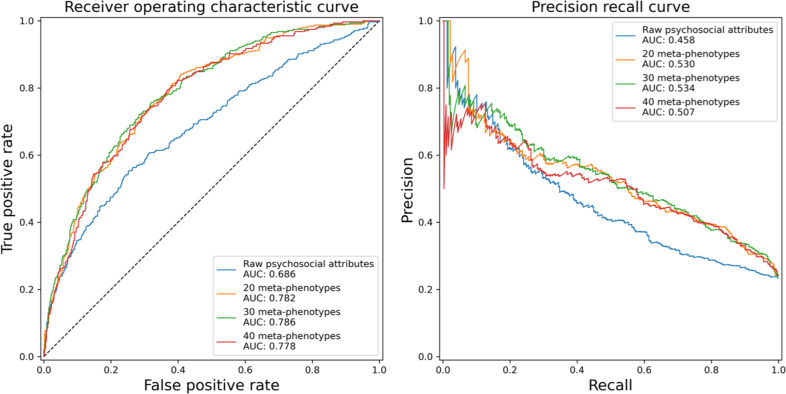
Classification accuracy of five-fold cross-validations in AUC curves and AUPRC (MixEHR meta-phenotypes vs. raw psychosocial attributes). AUC area under the receiver operating characteristic, AUPRC area under the precision-recall curve.

To evaluate the classification performances of meta-phenotypes and MDD diagnosis, we compared AUC and AUPRC for different MixEHR models and MDD diagnosis. We ran MixEHR models based on both the questionnaire data (clinical and psychosocial predictors) only and the questionnaire and SNP data. The MixEHR model with the questionnaire data provided better classification accuracy, so we focused on the MixEHR model using the questionnaire data only. Overall, the 30-meta-phenotype topic model outperformed other models and had the highest values (AUC = 0.786 and AUPRC = 0.534). Figure 3 shows the classification accuracy of 5-fold cross-validations in AUC and AUPRC for meta-phenotypes and the raw clinical and psychosocial predictors. The MixEHR-derived 30 meta-phenotypes had superior performance in both AUC and AUPRC.

### Genetic associations with MDD meta-phenotypes

**Fig. 4:**
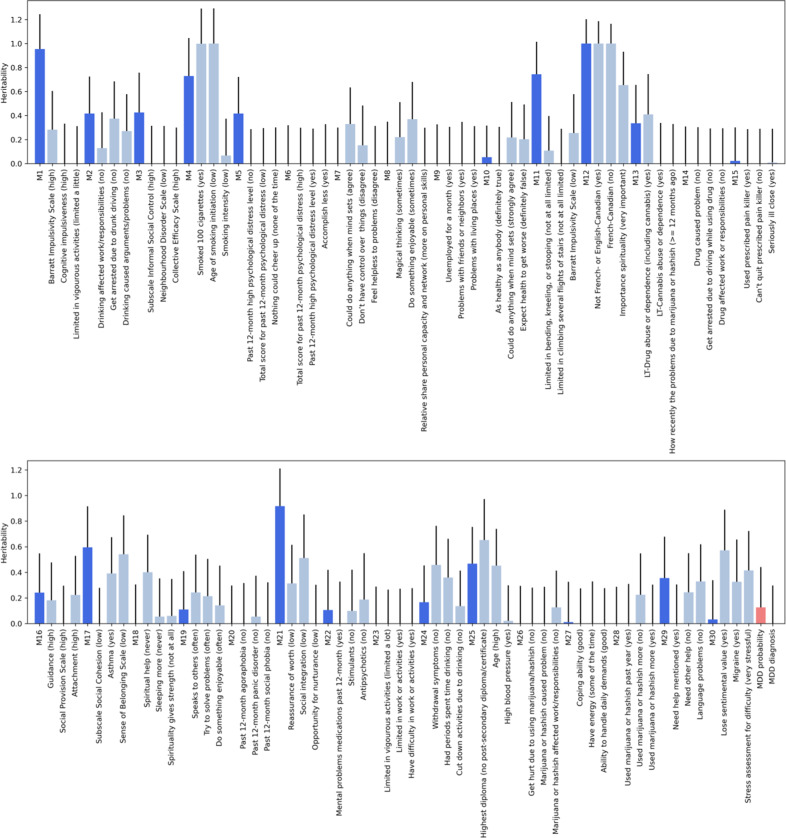
SNP-heritability for 30 meta-phenotypes and top three predictors for each meta-phenotype.

To further investigate whether MDD probability is related to the known neurobiological factors in the literature, we examined heritability for the 30 latent meta-phenotypes, MDD probability, and MDD diagnosis. Figure 4 illustrates the SNP-heritability estimates for 30 meta-phenotypes along with their top-3 predictors. We found that some meta-phenotypes (e.g., M1, M3, M5, M11, and M21) had higher heritability estimates than their top-3 predictors. The estimated SNP-heritability for MDD probability (estimate = 0.126, SE = 0.316) was much higher than the estimates for MDD diagnosis (estimate = 0.000001, SE = 0.297) although statistically non-significant due to large standard error likely because of the small sample size.

**Fig. 5:**
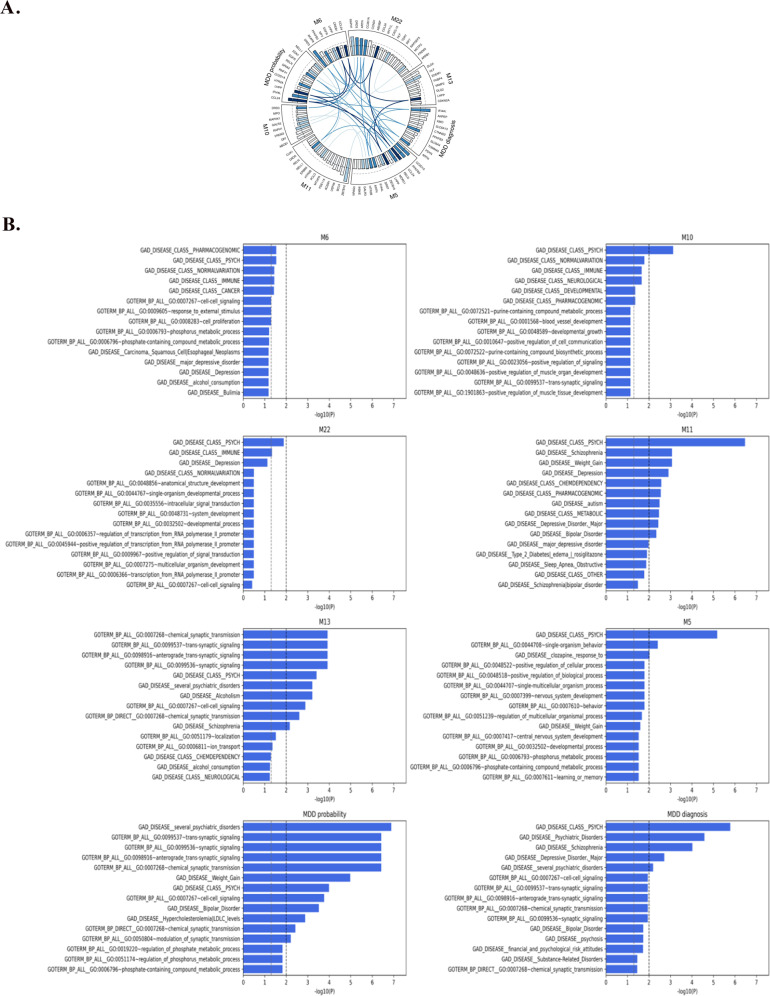
Pascal gene scores and MDD significant pathways associated with Top six meta-phenotypes, MDD probability, and MDD diagnosis. **A** Pascal gene scores for Top six meta-phenotypes, MDD probability, and MDD diagnosis, respectively; **B** MDD significant pathways associated with top six meta-phenotypes, MDD probability, and MDD diagnosis.

We then computed gene scores for MDD diagnosis, MDD probability, and the top six meta-phenotypes having the strongest correlations with MDD diagnosis, respectively, using the Pascal algorithm. Figure 5A provides a list of significant genes associated with MDD probability, MDD diagnosis, and the top six meta-phenotypes identified (see Fig. 2B for the list of top six meta-phenotypes). MDD probability and these top six meta-phenotypes identified several MDD associated genes that have been suggested in the literature, whereas MDD diagnosis was not associated with them, for instance, Dopamine Receptor D3 (DRD3), 6-phosphofructo-2-kinase/fructose-2,6-bisphosphatase (PFKFB3), Solute Carrier Family 6 Member 4 (SLC6A4), 5-Hydroxytryptamine Receptor 2 A (HTR2A), and Coiled-Coil And C2 Domain Containing 1 A (CC2D1A), that has been consistently proven its genetic predisposition for MDD. MDD diagnosis and MDD probability identified different sets of significant genes with one gene- Interferon Induced Protein 44 Like (IFI44L) identified by these two phenotypes.

Pathway enrichment analyses were conducted on those MDD significant genes. These selected meta-phenotypes were associated with different combinations of significant pathways, and both MDD probability and MDD diagnosis identified psychiatric disorders-related pathways. Figure 5B summarizes MDD significant pathways (including both those with *p* < 0.05 and *p* < 0.01) associated with each meta-phenotype, MDD probability, and MDD diagnosis in a Circos plot. A substantial number of genes were shared among the meta-phenotypes, MDD, and MDD probability, implying pleiotropy of causal architectures among these MDD-related traits.

We further explored the gene score correlation for top meta-phenotypes and found that only M6 was genetically correlated with M5, whereas other meta-phenotypes did not exhibit significant genetic correlations with each other. Fig. S1 provides the Pascal gene score correlation based on MDD-related genes.

## Discussion

The present study is one of the first analyses applying the topic model to categorize the risk of MDD using a wide range of clinical and psychosocial predictors and demonstrating the latent MDD phenotypes augmenting the capacity of exploring meaningful genetic variants that are linked with clinical subgroups of MDD. Our findings illustrate the adaptation of state-of-art technology – unsupervised machine learning –in improving prediction accuracy. Our findings also shed light on investigating and interpreting susceptible genetic predispositions in the etiopathogenesis of MDD.

Our study yielded two major findings. First, we identified the following six latent MDD phenotypes that confer the risk categorization of MDD including high psychological distress (M6), pre-existing substance abuse problems (M13), use of medications for mental health problems (M22), better self-perceived health (M10), limited restrictions on mobility and no/limited impulsiveness (M11), and no/low psychological distress (M5). Prior work on MDD risk categorizations has also identified some of these meta-phenotypes consistently associated with MDD: people with a high level of psychological distress, pre-existing substance abuse problems, and use of mental health medications were positively correlated with MDD, whereas those with better self-perceived health, low level of psychological distress, or limited restrictions on mobility less likely to have MDD [32–34].

High psychological distress predicts a high level of psychological stress. The vulnerability-stress model suggests that multiple risk factors throughout the development period interact with stressors and protective factors contributing to either normal development or psychopathology, such as MDD [35]. The cumulative effect of multiple stressors can produce lasting effects on the neural structure or function as well as stress physiology and increase the risk of MDD [36, 37]. Our data-driven approach with the focus on diversified clinical and psychosocial profiles detected a total of 30 latent meta-phenotypes that were associated with MDD diagnosis, especially the following three meta-phenotypes positively correlated with the risk of MDD, including stress-related subgroup (M6), comorbidity with substance abuse subgroup (M13), and the use of antidepressants in the past 12-month period prior to the data collection (M22). In clinical practice, MDD with co-occurring illicit drug and alcohol abuse or dependence has distinguishing characteristics, including family history, depressive symptomatology, suicidal ideation, and the treatment outcome of depression [38]. Antidepressants primarily work on the monoamine neurotransmission system to increase levels of serotonin, norepinephrine, and/or dopamine by alleviating depressive symptoms [39]. Our latent model identified a group of depressed patients on antidepressants indicating a group of depressed patients fall within a more homogeneous “monoamine neurotransmitter subtype” that is attributed to the functional imbalance or deficiency of monoamine-series neurotransmitters, including dopamine, serotonin, and norepinephrine [40].

The second major finding of the present study is that genetic predispositions (including susceptible genes and pathways) were identified by these latent meta-phenotypes and MDD probability derived from these latent meta-phenotypes. Compared to the questionnaire-derived MDD diagnosis, some latent meta-phenotypes (e.g., M1, M3, M5, M11, and M21) had higher heritability and MDD probability and its meta-phenotypes detected a list of significant MDD-related genes and pathways. Some of these genes, including DRD3, PFKFB3, SLC6A4, HTR2A, are involved in the different etiological hypotheses of MDD, including *the monoamine theory* (i.e., SLC6A4, which encodes the serotonin transporter that is responsible for the reuptake of serotonin; HTR2A, receptor genes for serotonin), *the stress-induced theory* (i.e., FKBP Prolyl Isomerase 5 (FKBP5), one of stress hormone genes, one of a key player in human response to stress), *the cytokine and inflammatory response hypothesis* (i.e. C-C Motif Chemokine Ligand 24 (CCL24), a part of the subfamily of small cytokine genes, proinflammatory cytokines contribute to the major symptoms of MDD; IFI44L, belongs to interferon-induced protein family with inflammatory function), *circadian rhythm disturbances* (i.e., A-Kinase Anchoring Protein 8 (AKAP8), involved in the biosynthesis of the circadian hormone melatonin), and *the neurodevelopmental theory* (i.e., Ras Association (RalGDS/AF-6) And Pleckstrin Homology Domains 1 (RAPH1), responsible for proper neuronal migration) [41–43].

Our pathway enrichment analyses also diversified pathways related to different functioning, including psychiatric disorders, basic cellular functioning, immune, the development of cancer, neurological and developmental functioning. For instance, the high psychological distress (M6) subgroup identified top pathways involved in the occurrence of psychiatric disorders, including MDD, as well as a pharmacogenomics-based pathway, that is involved in pharmacokinetic and pharmacodynamic processes [44]. Comorbid MDD and substance use subgroup (M13) identified more essential pathways related to synapse formation, for instance, chemical-synaptic-transmission, and synaptic signaling. Synapse formation plays an important role in neurocircuitry and likely requires interactions between pre-and post-synaptic neurons [45]. The use of antidepressants subgroup (M22) found the top significant pathways related to psychiatric disorders, immune functioning, and depression. The better self-rated health subgroup (M10) identified pathways more related to psychiatric disorders, immune functioning, neurological and developmental pathways. Limited restrictions on mobility and no/limited impulsiveness (M11) identified pathways related to psychiatric disorders, schizophrenia, depression, and weight gain. No/low psychological distress (M5) identified pathways related to psychiatric disorders, single-organism behavior, response to clozapine, and positive regulation of the cellular process. MDD probability identified pathways related to psychiatric disorders, synapse formation, and cell-cell signaling.

Blano-Gomez et al. believed that the complex origins of MDD to some extent result from a component of multiple traits with polygenic influence [46]. This present study further expands the literature by applying a multivariate modeling process to detect key predictors associated with the risk of MDD and its subtypes. These predictors and the MDD phenotypes derived from these predictors not only discover known MDD genes and pathways but also provide evidence to support the multiple causes connected to different pathogenesis and heterogeneous clinical manifestations.

The findings of the present study have several clinical and research implications. First, these findings suggest a holistic approach to settle the long-debated nosology of MDD that can be further categorized into subtypes with stress-related and those with abnormal neurobiological changes [47, 48]. Second, the most significant MDD meta-phenotypes identified could help to identify MDD subtypes and suggest appropriate clinical management strategies for different subtypes. Third, our findings direct future research to focus on these identified genes and pathways for MDD subtypes.

The present study adopted a latent topic model and utilized rich, well-characterized clinical and psychosocial predictors to identify latent MDD meta-phenotypes and categorize MDD. The study cohort consists of community-based samples, which are from the general population. The generalizability of the research findings is excellent. Although the latent topic model is a data-driven approach, it does identify biologically meaningful genetic variants that are known for their biological functioning.

Three limitations should be noted. First, we are not aware of any population-based cohorts with rich psychosocial information to replicate our current findings. Our results should be taken as exploratory, although their plausibility is supported by previous research and cross-validation. Second, our power to detect genetic variants is limited, which restricts us to run the latent topic model with the biopsychosocial framework of MDD. Third, our psychosocial data are self-reported and hence subject to information biases.

Overall, our risk prediction model confers not only accurate MDD risk categorization but also meaningful associations with genetic predispositions that are linked to MDD subtypes. The findings of the present study could help to navigate appropriate clinical management strategies for various MDD subtypes and support multidisciplinary risk prediction research by benefiting the state-of-art technology– unsupervised machine learning algorithms.

## Supplementary information

Supplemental materials

## Data Availability

The code used in this study is available from the authors upon reasonable request.
