# Supplementary material for: Integrative PheWAS analysis in risk categorization of major depressive disorder and identifying their associations with genetic variants using a latent topic model approach
Source: Transl Psychiatry. 2022 Jun 8;12:240. doi: 10.1038/s41398-022-02015-8 (PMC9177831; doi:10.1038/s41398-022-02015-8)

**Integrative PheWAS analysis in risk categorization of major depressive disorder and identifying their associations with genetic variants using a latent topic model approach**

Xiangfei Meng, Michelle Wang, Kieran J. O’Donnell, Jean Caron, Michael J. Meaney, Yue Li

**Corresponding author:**

Correspondence to Xiangfei Meng & Yue Li.

Email: [xiangfei.meng@mcgill.ca](mailto:xiangfei.meng@mcgill.ca) & [yueli@cs.mcgill.ca](mailto:yueli@cs.mcgill.ca)

**SUPPLEMENTARY INFORMATION**

**Table S1**. A list of abbreviations and their explanations

**Table S2**. A full list of variables analyzed in the present study

**Figure S1**. Pascal gene scores comparison between MDD probability and MDD diagnosis

**Table S1**. A list of abbreviations and their explanations

| **Abbreviations** | **Explanations** |
| --- | --- |
| MDD | Major depressive disorder |
| GWAS | Genome-wide association studies |
| TRIPOD | Transparent Reporting of a Multivariable Prediction Model for Individual Prognosis or Diagnosis |
| ZEPSOM | Zone d'Épidémiologie Psychiatrique du Sud-Ouest de Montréal |
| WHO-CIDI | World Health Organization version of Composite International Diagnostic Interview |
| DSM-IV | Diagnostic and Statistical Manual of Mental Disorders |
| ICD-10 | International Statistical Classification of Diseases and Related Health Problems, 10^th^ revision |
| SNP | Single-nucleotide polymorphism |
| LDA | Latent Dirichlet Allocation |
| LASSO | Least absolute shrinkage and selection operator |
| AUC | Area under the receiver operating characteristic |
| AUPRC | Area under the precision-recall curve |
| MAF | Minor allele frequency |
| REML | Restricted maximum likelihood algorithm |
| DRD3 | Dopamine Receptor D3 |
| PFKFB3 | 6-phosphofructo-2-kinase/fructose-2,6-bisphosphatase |
| SLC6A4 | Solute Carrier Family 6 Member 4 |
| HTR2A | 5-Hydroxytryptamine Receptor 2A |
| CC2D1A | Coiled-Coil and C2 Domain Containing 1A |
| IFI44L | Interferon Induced Protein 44 Like |
| FKBP5 | FKBP Prolyl Isomerase 5 |
| CCL24 | C-C Motif Chemokine Ligand 24 |
| AKAP8 | A-Kinase Anchoring Protein 8 |
| RAPH1 | Ras Association (RalGDS/AF-6) And Pleckstrin Homology Domains 1 |

**Table S2**. A full list of variables analyzed in the present study

| # | Description | Category | Abbreviations |
| --- | --- | --- | --- |
| 1 | Dimensions of quality of life (QoL) from the Satisfaction with Life Domains scale | Quality of life | Housing-neighbourhood |
| 2 | Dimensions of quality of life (QoL) from the Satisfaction with Life Domains scale | Quality of life | Social relations |
| 3 | Dimensions of quality of life (QoL) from the Satisfaction with Life Domains scale | Quality of life | Personal relationships |
| 4 | Dimensions of quality of life (QoL) from the Satisfaction with Life Domains scale | Quality of life | Autonomy |
| 5 | Dimensions of quality of life (QoL) from the Satisfaction with Life Domains scale | Quality of life | Spare time activities |
| 6 | Total score of quality of life from the Satisfaction with Life Domains scale | Quality of life | Quality of Life |
| 7 | Total score of mental well-being based on the Mental Health Continuum Short Form | Mental well-being | Mental well-being |
| 8 | Categorical measure of Mental well-being | Mental well-being | Categorical measure of Mental well-being |
| 9 | Personal well-being Index | Mental well-being | Personal well-being Index |
| 10 | National well-being Index | Mental well-being | National well-being Index |
| 11 | Total score of community participation based on the Community Involvement Scale | Participant’s perception of their neighborhood | Community Participant Scale |
| 12 | Total Resident Disempowerment based on the Resident Disempowerment Scale | Participant’s perception of their neighborhood | Resident Disempowerment Scale |
| 13 | Total score of neighborhood Disorder based on the Neighborhood Disorder Scale | Participant’s perception of their neighborhood | Neighborhood Disorder Scale |
| 14 | Total score of sense of collective efficacy based on the Sense of Collective Efficacy scale | Participant’s perception of their neighborhood | Collective Efficacy |
| 15 | Total score of informal social control based on the Neighbouring Behaviour Scale | Participant’s perception of their neighborhood | Subscale Informal Social Control |
| 16 | Total score of social cohesion based on the Neighbouring Behaviour Scale | Participant’s perception of their neighborhood | Subscale Social Cohesion |
| 17 | Total score of physical conditions based on the Neighbouring Behaviour Scale | Participant’s perception of their neighborhood | Physical Conditions |
| 18 | Total score of perceived safety scale based on the Neighbouring Behaviour Scale | Participant’s perception of their neighborhood | Perceived Safety |
| 19 | Total score of informal social control based on the Neighbouring Behaviour Scale | Participant’s perception of their neighborhood | Neighbouring Behaviour Scale |
| 20 | Total score of informal social control based on the Sense of belonging scale | Participant’s perception of their neighborhood | Sense of Belonging Scale |
| 21 | Dimensions of social support measured by the Social Provision Scale | Social support | Tangible help |
| 22 | Dimensions of social support measured by the Social Provision Scale | Social support | Attachment |
| 23 | Dimensions of social support measured by the Social Provision scale | Social support | Social integration |
| 24 | Dimensions of social support measured by the Social Provision scale | Social support | Reassurance of worth |
| 25 | Dimensions of social support measured by the Social Provision scale | Social support | Guidance |
| 26 | Dimensions of social support measured by the Social Provision scale | Social support | Opportunity for nurturance |
|  |  |  |  |
| 27 | Total score of the Social Provision Scale | Social support | Total score of social support |
| 28 | Total score of impulsiveness measured by the Barratt Impulsivity Scale | Impulsivity | Barratt Impulsivity Scale |
| 29 | Dimensions of impulsiveness of impulsivity measured by the Barratt Impulsivity Scale | Impulsivity | Non-planning impulsiveness |
| 30 | Dimensions of impulsiveness of impulsivity measured by the Barratt Impulsivity Scale | Impulsivity | Cognitive impulsiveness |
| 31 | Dimensions of impulsiveness of impulsivity measured by the Barratt Impulsivity Scale | Impulsivity | Motor impulsiveness |
| 32 | Total score of the Montreal Cognitive Assessment | Cognitive Impairment | Montreal Cognitive Assessment |
| 33 | Item of Pearlin’s Mastery Scale | Mastery | Feel unable to solve problems |
| 34 | Item of Pearlin’s Mastery Scale | Mastery | Feel shaken life |
| 35 | Item of Pearlin’s Mastery Scale | Mastery | Don’t have control over things |
| 36 | Item of Pearlin’s Mastery Scale | Mastery | Could do anything when mind sets seriously |
| 37 | Item of Pearlin’s Mastery Scale | Mastery | Feel helpless to problems |
| 38 | Item of Pearlin’s Mastery Scale | Mastery | What happens depends on me |
| 39 | Item of Pearlin’s Mastery Scale | Mastery | Little I can do to change |
| 40 | Comments of the Pearlin’s Mastery Scale | Mastery | Comments of the Pearlin’s Mastery Scale |
| 41 | Functional Disability measured by WHO Disability Assessment Schedule | Functional Disability | Functional Disability |
| 42 | Diagnosed of posttraumatic stress disorder during the past 12-months? | PTSD | Diagnosis of posttraumatic stress disorder |
| 43 | Item of the Perception of Services Needs | Perception of Services Needs | Ever received information help during the past 12-months prior to the interview? |
| 44 | Item of the Perception of Services Needs | Perception of Services Needs | Ever received medication help during the past 12-months prior to the interview? |
| 45 | Item of the Perception of Services Needs | Perception of Services Needs | Ever received counselling help during the past 12-months prior to the interview? |
| 46 | Item of the Perception of Services Needs | Perception of Services Needs | Ever received other help during the past 12-months prior to the interview? |
| 47 | Item of the Perception of Services Needs | Perception of Services Needs | Specify other help during the past 12-months prior to the interview? |
| 48 | Item of the Perception of Services Needs | Perception of Services Needs | Received adequate help during the past 12-months prior to the interview? |
| 49 | Item of the Perception of Services Needs | Perception of Services Needs | Need more information during the past 12-months prior to the interview? |
| 50 | Item of the Perception of Services Needs | Perception of Services Needs | Need more medication during the past 12-months prior to the interview? |
| 51 | Item of the Perception of Services Needs | Perception of Services Needs | Need more counselling during the past 12-months prior to the interview? |
| 52 | Item of the Perception of Services Needs | Perception of Services Needs | Need other help during the past 12-months prior to the interview? |
| 53 | Item of the Perception of Services Needs | Perception of Services Needs | Why didn’t you get more information about these problems, treatments or available services (during the past 12 months)?  Manage yourself? |
| 54 | Item of the Perception of Services Needs | Perception of Services Needs | Why didn’t you get more information about these problems, treatments or available services (during the past 12 months)?  Nowhere to get? |
| 55 | Item of the Perception of Services Needs | Perception of Services Needs | Why didn’t you get more information about these problems, treatments or available services (during the past 12 months)?  No time? |
| 56 | Item of the Perception of Services Needs | Perception of Services Needs | Why didn’t you get more information about these problems, treatments or available services (during the past 12 months)?  Job interfered? |
| 57 | Item of the Perception of Services Needs | Perception of Services Needs | Why didn’t you get more information about these problems, treatments or available services (during the past 12 months)?  Not available? |
| 58 | Item of the Perception of Services Needs | Perception of Services Needs | Why didn’t you get more information about these problems, treatments or available services (during the past 12 months)?  No confidence in health services? |
| 59 | Item of the Perception of Services Needs | Perception of Services Needs | Why didn’t you get more information about these problems, treatments or available services (during the past 12 months)?  Not affordable? |
| 60 | Item of the Perception of Services Needs | Perception of Services Needs | Why didn’t you get more information about these problems, treatments or available services (during the past 12 months)?  No insurance coverage? |
| 61 | Item of the Perception of Services Needs | Perception of Services Needs | Why didn’t you get more information about these problems, treatments or available services (during the past 12 months)?  Be afraid of others’ thoughts? |
| 62 | Item of the Perception of Services Needs | Perception of Services Needs | Why didn’t you get more information about these problems, treatments or available services (during the past 12 months)?  Language problems? |
| 63 | Item of the Perception of Services Needs | Perception of Services Needs | Why didn’t you get more information about these problems, treatments or available services (during the past 12 months)?  Help is ongoing? |
| 64 | Item of the Perception of Services Needs | Perception of Services Needs | Why didn’t you get more information about these problems, treatments or available services (during the past 12 months)?  Other? |
| 65 | Item of the Perception of Services Needs | Perception of Services Needs | Why didn’t you get more information about these problems, treatments or available services (during the past 12 months)?  Specify other reasons |
| 66 | Item of the Perception of Services Needs | Perception of Services Needs | Didn't receive more information - Reason for preferring to manage yourself  You think people don't know how to help, because of? |
| 67 | Item of the Perception of Services Needs | Perception of Services Needs | Didn't receive more information - Reason for preferring to manage yourself  Reason for managing yourself, please specify |
| 68 | Item of the Perception of Services Needs | Perception of Services Needs | Didn't receive more medication - Manage yourself? |
| 69 | Item of the Perception of Services Needs | Perception of Services Needs | Didn't receive more medication - Nowhere to get? |
| 70 | Item of the Perception of Services Needs | Perception of Services Needs | Didn't receive more medication - No time? |
| 71 | Item of the Perception of Services Needs | Perception of Services Needs | Didn't receive more medication - Job interfered? |
| 72 | Item of the Perception of Services Needs | Perception of Services Needs | Didn't receive more medication - Not available? |
| 73 | Item of the Perception of Services Needs | Perception of Services Needs | Didn't receive more medication - No confidence in health services? |
| 74 | Item of the Perception of Services Needs | Perception of Services Needs | Didn't receive more medication - Not affordable? |
| 75 | Item of the Perception of Services Needs | Perception of Services Needs | Didn't receive more medication - No insurance coverage? |
| 76 | Item of the Perception of Services Needs | Perception of Services Needs | Didn't receive more medication - Be afraid of others’ thoughts? |
| 77 | Item of the Perception of Services Needs | Perception of Services Needs | Didn't receive more medication - Language problems? |
| 78 | Item of the Perception of Services Needs | Perception of Services Needs | Didn't receive more medication -Help is ongoing? |
| 79 | Item of the Perception of Services Needs | Perception of Services Needs | Didn't receive more medication – Other? |
| 80 | Item of the Perception of Services Needs | Perception of Services Needs | Didn't receive more medication - Specify other reasons? |
| 81 | Item of the Perception of Services Needs | Perception of Services Needs | Didn't receive more medication -Reason for managing yourself? |
| 82 | Item of the Perception of Services Needs | Perception of Services Needs | Didn't receive more medication -Specify reason for managing yourself? |
| 83 | Item of the Perception of Services Needs | Perception of Services Needs | Didn't receive more consultation therapy or help, prefer to manage yourself? |
| 84 | Item of the Perception of Services Needs | Perception of Services Needs | Didn't receive more consultation therapy or help, nowhere to get? |
| 85 | Item of the Perception of Services Needs | Perception of Services Needs | Didn't receive more consultation therapy or help, because no time |
| 86 | Item of the Perception of Services Needs | Perception of Services Needs | Didn't receive more consultation therapy or help, job interfered? |
| 87 | Item of the Perception of Services Needs | Perception of Services Needs | Didn't receive more consultation therapy or help, the help is not available? |
| 88 | Item of the Perception of Services Needs | Perception of Services Needs | Didn't receive more consultation therapy or help, you have no confidence in health services? |
| 89 | Item of the Perception of Services Needs | Perception of Services Needs | Didn't receive more consultation therapy or help, the help is not affordable? |
| 90 | Item of the Perception of Services Needs | Perception of Services Needs | Didn't receive more consultation therapy or help, you have no insurance coverage |
| 91 | Item of the Perception of Services Needs | Perception of Services Needs | Didn't receive more consultation therapy or help, you are afraid of others’ thoughts |
| 92 | Item of the Perception of Services Needs | Perception of Services Needs | Didn't receive more consultation therapy or help, language problems? |
| 93 | Item of the Perception of Services Needs | Perception of Services Needs | Didn't receive more consultation therapy or help, help is ongoing? |
| 94 | Item of the Perception of Services Needs | Perception of Services Needs | Didn't receive more consultation therapy or help, other reasons? |
| 95 | Item of the Perception of Services Needs | Perception of Services Needs | Didn't receive more consultation therapy or help. Specify the reason. |
| 96 | Item of the Perception of Services Needs | Perception of Services Needs | Didn't receive more counselling. Reason for managing yourself? |
| 97 | Item of the Perception of Services Needs | Perception of Services Needs | Didn't receive more counselling. Reason for preferring to manage by yourself. Specify. |
| 98 | Item of the Perception of Services Needs | Perception of Services Needs | Didn't receive more other help. Because you prefer to manage yourself? |
| 99 | Item of the Perception of Services Needs | Perception of Services Needs | Didn't receive more other help. Because you have nowhere to get? |
| 100 | Item of the Perception of Services Needs | Perception of Services Needs | Didn't receive more other help. Because you have no time? |
| 101 | Item of the Perception of Services Needs | Perception of Services Needs | Didn't receive more other help. Because job interfered? |
| 102 | Item of the Perception of Services Needs | Perception of Services Needs | Didn't receive more other help. Because you are not available? |
| 103 | Item of the Perception of Services Needs | Perception of Services Needs | Didn't receive more other help. Because you have no confidence in health services? |
| 104 | Item of the Perception of Services Needs | Perception of Services Needs | Didn't receive more other help. Because you cannot afford? |
| 105 | Item of the Perception of Services Needs | Perception of Services Needs | Didn't receive more other help. Because you have no insurance coverage? |
| 106 | Item of the Perception of Services Needs | Perception of Services Needs | Didn't receive more other help. Because you are afraid of others’ thoughts? |
| 107 | Item of the Perception of Services Needs | Perception of Services Needs | Didn't receive more other help. Because language problems? |
| 108 | Item of the Perception of Services Needs | Perception of Services Needs | Didn't receive more other help. Because have help ongoing? |
| 109 | Item of the Perception of Services Needs | Perception of Services Needs | Didn't receive more other help. Because other reasons? |
| 110 | Item of the Perception of Services Needs | Perception of Services Needs | Didn't receive more other help. Please specify other reasons |
| 111 | Item of the Perception of Services Needs | Perception of Services Needs | Didn't receive more other help. Reason for managing yourself? |
| 112 | Item of the Perception of Services Needs | Perception of Services Needs | Didn't receive more other help. Specify reason for managing yourself |
| 113 | Item of the Perception of Services Needs | Perception of Services Needs | You have some help needed? |
| 114 | Item of the Perception of Services Needs | Perception of Services Needs | Did you need information help? |
| 115 | Item of the Perception of Services Needs | Perception of Services Needs | Did you need medication help? |
| 116 | Item of the Perception of Services Needs | Perception of Services Needs | Did you need counselling help? |
| 117 | Item of the Perception of Services Needs | Perception of Services Needs | Did you need other help? |
| 118 | Item of the Perception of Services Needs | Perception of Services Needs | Reason for not receiving information help-Manage yourself? |
| 119 | Item of the Perception of Services Needs | Perception of Services Needs | Reason for not receiving information help-Nowhere to get? |
| 120 | Item of the Perception of Services Needs | Perception of Services Needs | Reason for not receiving information help -No time? |
| 121 | Item of the Perception of Services Needs | Perception of Services Needs | Reason for not receiving information help-Job interfered? |
| 122 | Item of the Perception of Services Needs | Perception of Services Needs | Reason for not receiving information help-Not available? |
| 123 | Item of the Perception of Services Needs | Perception of Services Needs | Reason for not receiving information help-No confidence in health services? |
| 124 | Item of the Perception of Services Needs | Perception of Services Needs | Reason for not receiving information help-Not affordable? |
| 125 | Item of the Perception of Services Needs | Perception of Services Needs | Reason for not receiving information help-No insurance coverage? |
| 126 | Item of the Perception of Services Needs | Perception of Services Needs | Reason for not receiving information help- Be afraid of others’ thoughts? |
| 127 | Item of the Perception of Services Needs | Perception of Services Needs | Reason for not receiving information help-Language problems? |
| 128 | Item of the Perception of Services Needs | Perception of Services Needs | Reason for not receiving information help-Other reasons? |
| 129 | Item of the Perception of Services Needs | Perception of Services Needs | Reason for not receiving information help-Specify other reasons |
| 130 | Item of the Perception of Services Needs | Perception of Services Needs | Did not receive medication- Reason for preferring to manage yourself? |
| 131 | Item of the Perception of Services Needs | Perception of Services Needs | Did not receive medication- Reason for preferring to manage yourself. Specify reason for managing yourself? |
| 132 | Item of the Perception of Services Needs | Perception of Services Needs | Reason for not receiving medication help- Manage yourself? |
| 133 | Item of the Perception of Services Needs | Perception of Services Needs | Reason for not receiving medication help- Nowhere to get? |
| 134 | Item of the Perception of Services Needs | Perception of Services Needs | Reason for not receiving medication help- No time? |
| 135 | Item of the Perception of Services Needs | Perception of Services Needs | Reason for not receiving medication help- Job interfered? |
| 136 | Item of the Perception of Services Needs | Perception of Services Needs | Reason for not receiving medication help- Not available? |
| 137 | Item of the Perception of Services Needs | Perception of Services Needs | Reason for not receiving medication help- No confidence in health services? |
| 138 | Item of the Perception of Services Needs | Perception of Services Needs | Reason for not receiving medication help- Not affordable? |
| 139 | Item of the Perception of Services Needs | Perception of Services Needs | Reason for not receiving medication help- No insurance coverage? |
| 140 | Item of the Perception of Services Needs | Perception of Services Needs | Reason for not receiving medication help- Be afraid of others’ thoughts? |
| 141 | Item of the Perception of Services Needs | Perception of Services Needs | Reason for not receiving medication help- Language problems |
| 142 | Item of the Perception of Services Needs | Perception of Services Needs | Reason for not receiving medication help- Other reasons? |
| 143 | Item of the Perception of Services Needs | Perception of Services Needs | Reason for not receiving medication help- Specify other reasons? |
| 144 | Item of the Perception of Services Needs | Perception of Services Needs | Reason for not receiving medication help- Reason for managing yourself? |
| 145 | Item of the Perception of Services Needs | Perception of Services Needs | Reason for not receiving medication help- Specify reason for managing yourself. |
| 146 | Item of the Perception of Services Needs | Perception of Services Needs | Reason for not receiving counselling help- Manage yourself? |
| 147 | Item of the Perception of Services Needs | Perception of Services Needs | Reason for not receiving counselling help- Nowhere to get? |
| 148 | Item of the Perception of Services Needs | Perception of Services Needs | Reason for not receiving counselling help- No time? |
| 149 | Item of the Perception of Services Needs | Perception of Services Needs | Reason for not receiving counselling help- Job interfered? |
| 150 | Item of the Perception of Services Needs | Perception of Services Needs | Reason for not receiving counselling help- Not available? |
| 151 | Item of the Perception of Services Needs | Perception of Services Needs | Reason for not receiving counselling help- No confidence in health services? |
| 152 | Item of the Perception of Services Needs | Perception of Services Needs | Reason for not receiving counselling help- Not affordable? |
| 153 | Item of the Perception of Services Needs | Perception of Services Needs | Reason for not receiving counselling help- No insurance coverage? |
| 154 | Item of the Perception of Services Needs | Perception of Services Needs | Reason for not receiving counselling help- Be afraid of others’ thoughts? |
| 155 | Item of the Perception of Services Needs | Perception of Services Needs | Reason for not receiving counselling help- Language problems? |
| 156 | Item of the Perception of Services Needs | Perception of Services Needs | Reason for not receiving counselling help- Other reasons? |
| 157 | Item of the Perception of Services Needs | Perception of Services Needs | Reason for not receiving counselling help- Specify other reasons? |
| 158 | Item of the Perception of Services Needs | Perception of Services Needs | Reason for not receiving counselling help- Reason for managing yourself? |
| 159 | Item of the Perception of Services Needs | Perception of Services Needs | Reason for not receiving counselling help- Specify reason for managing yourself? |
| 160 | Item of the Perception of Services Needs | Perception of Services Needs | Commentaries of the Perception of Services Needs |
| 161 | Gambling measured by the Canadian Problem Gambling Index | Gambling | Canadian Problem Gambling Index |
| 162 | Dimensions of self-reported aggressive behavior measured by the Modified Overt Aggression Scale | Self-reported aggressive behavior | Verbal aggression |
| 163 | Dimensions of self-reported aggressive behavior measured by the Modified Overt Aggression Scale | Self-reported aggressive behavior | Aggression against property |
| 164 | Dimensions of self-reported aggressive behavior measured by the Modified Overt Aggression Scale | Self-reported aggressive behavior | Aggression against self |
| 165 | Dimensions of self-reported aggressive behavior measured by the Modified Overt Aggression Scale | Self-reported aggressive behavior | Physical aggression |
| 166 | Has a doctor ever told you that you have diabetes? | Physical Health measures | Diabetes |
| 167 | How old were you when diabetes was first diagnosed? | Physical Health measures | Age onset of diabetes |
| 168 | How long was it before you started insulin? | Physical Health measures | Diabetes duration |
| 169 | Do you currently take insulin? | Physical Health measures | Whether take insulin |
| 170 | Has a doctor ever told you that you have asthma? | Physical Health measures | Asthma |
| 171 | How old were you when asthma was first diagnosed? | Physical Health measures | Age onset of asthma |
| 172 | Did you take asthma medications such as inhalers or nebulizers? | Physical Health measures | Take asthma medication |
| 173 | Has a doctor ever told you that you have hypertension? | Physical Health measures | Hypertension |
| 174 | How old were you when hypertension was first diagnosed? | Physical Health measures | Age onset of hypertension |
| 175 | Did you take medications for hypertension? | Physical Health measures | Take medication for hypertension |
| 176 | Has a doctor ever told you that you have heart disease? | Physical Health measures | Heart disease |
| 177 | How old were you when heart disease was first diagnosed? | Physical Health measures | Age onset of heart disease |
| 178 | Did you take medications for heart disease? | Physical Health measures | Take medication for heart disease |
| 179 | Has a doctor ever told you that you have stomach or intestinal ulcers? | Physical Health measures | Stomach or intestinal ulcers |
| 180 | How old were you when stomach or intestinal ulcers was first diagnosed? | Physical Health measures | Age onset of stomach or intestinal ulcers |
| 181 | Has a doctor ever told you that you have arthritis/rheumatism? | Physical Health measures | Arthritis/rheumatism |
| 182 | How old were you when arthritis/rheumatism was first diagnosed? | Physical Health measures | Age onset of arthritis/rheumatism |
| 183 | Has a doctor ever told you that you have migraine headaches? | Physical Health measures | Migraine |
| 184 | How old were you when migraine headaches were first diagnosed? | Physical Health measures | Age onset of migraine |
| 185 | Has a doctor ever told you that you have cancer? | Physical Health measures | Cancer |
| 186 | How old were you when cancer was first diagnosed? | Physical Health measures | Age onset of cancer |
| 187 | Has a doctor ever told you that you have kidney disease? | Physical Health measures | Kidney disease |
| 188 | How old were you when kidney disease was first diagnosed? | Physical Health measures | Age onset of kidney disease |
| 189 | Has a doctor ever told you that you have a back problem? | Physical Health measures | Back problems |
| 190 | How old were you when the back problem was first diagnosed? | Physical Health measures | Age onset of back problems |
| 191 | Did you take pain relievers for the back problem? | Physical Health measures | Take pain relievers |
| 192 | Did you take antidepressants for hypertension? | Physical Health measures | Take anti-depressants |
| 193 | Hours of sleep per day | Sleep | Hours of sleep per day |
| 194 | Minutes of sleep per day | Sleep | Minutes of sleep per day |
| 195 | Quality of sleep | Sleep | Quality of sleep |
| 196 | Number of days per week worked at least 2 hours from 10 pm to 5 am | Sleep | N of days per week worked at least 2 hours from 10 pm to 5 am |
| 197 | Would you say that in general, your health is? | Healthy Days Core Module | Health status |
| 198 | How many days was your physical health not good in the past 30 days? | Healthy Days Core Module | Physical health not good days |
| 199 | Now thinking about your mental health, which includes stress, depression, and problems with emotions, for how many days during the past 30 days was your mental health not good? | Healthy Days Core Module | Mental health not good days |
| 200 | During the past 30 days, for about how many days did poor physical or mental health keep you from doing your usual activities, such as self-care, work, or recreation? | Healthy Days Core Module | Inactive days |
| 201 | Compared to one year ago, how would you rate your general health now? | Healthy Days Core Module | General health rating |
| 202 | In your lifetime, have you smoked a total of 100 or more cigarettes? | Smoking | Ever smoked 100 cigarettes |
| 203 | Do you smoke cigarettes? | Smoking | Present cigarette smoking |
| 204 | At what age did you smoke you first whole cigarette? | Smoking | Age of smoking initiation |
| 205 | On average, how many cigarettes do you smoke each day? | Smoking | Smoking intensity |
| 206 | Does your health limit you in any of the following activities: in vigorous activities, such as running, lifting heavy objects, or participating in strenuous sports? | Functional disability | Limited in vigorous activities |
| 207 | Limited in moderate activities  in moderate activities, such as moving a table, pushing a vacuum cleaner, bowling, or playing golf? | Functional disability | Limited in moderate activities |
| 208 | Limited in lifting or carrying groceries  in lifting or carrying groceries? | Functional disability | Limited in lifting groceries |
| 209 | Limited in climbing several flights of stairs  in climbing several flights of stairs? | Functional disability | Limited in climbing several flights of stairs |
| 210 | Limited in climbing one flight of stairs  In climbing one flight of stairs? | Functional disability | Limited in climbing one flight of stairs |
| 211 | Limited in bending, kneeling, or stooping  In bending, kneeling, or stooping? | Functional disability | Limited in bending, kneeling, or stooping |
| 212 | Limited in walking more than one kilometer  In walking more than one kilometre? | Functional disability | Limited in walking more than 1KM |
| 213 | Limited in walking several blocks  In walking several blocks? | Functional disability | Limited in walking several blocks |
| 214 | Limited in walking one block  In walking one block? | Functional disability | Limited in walking one block |
| 215 | Limited in bathing and dressing yourself  In bathing and dressing yourself? | Functional disability | Limited in bathing and dressing |
| 216 | Cut down activities due to physical problems  Because of your physical health, during the past 4 weeks, did you: cut down on the amount of time you spent on work or other activities? | Functional disability | Cut down activities |
| 217 | Accomplish less than you would like due to physical problem  Accomplish less than you would like? | Functional disability | Accomplish less |
| 218 | Limited in the kind of work or other activities due to physical problem  Limited in the kind of work you do or other activities? | Functional disability | Limited in work or activities |
| 219 | Have difficulty performing the work or other activities due to physical problem  Have difficulty performing the work you do or other activities (for example, it took extra effort)? | Functional disability | Have difficulty in work or activities |
| 220 | Cut down activities because of emotional problems  Because of emotional problems during the past 4 weeks, did you:  cut down on the amount of time you spent on work or other activities? | Functional disability | Cut down activities |
| 221 | Accomplish less than you would like because of emotional problems  Accomplish less than you would like? | Functional disability | Accomplish less |
| 222 | Not do work or other activities as carefully as usual due to emotional problems  Not do work or other activities as carefully as usual? | Functional disability | Not do work or activities carefully |
| 223 | Physical or emotional problems interfered with normal social activities  During the past 4 weeks, how much has your physical health or emotional problems interfered with your normal social activities with family, friends, neighbours, or groups? | Functional disability | Physical/emotional problem affected social activities |
| 224 | Bodily pain in the past 4 weeks  During the past 4 weeks, how much bodily pain have you had? | Functional disability | Bodily pain |
| 225 | Pain interfered with normal work  During the past 4 weeks, how much did pain interfere with your normal work (including work both outside the home and housework)? | Functional disability | Pain affected work |
| 226 | During the past 4 weeks, how much of the time:  did you feel full of pep? | Patient Health Questionnaire | Feel full of pep |
| 227 | Have you been a very nervous person? | Patient Health Questionnaire | Been very nervous |
| 228 | Have you felt so down in the dumps that nothing could cheer you up? | Patient Health Questionnaire | Nothing could cheer up |
| 229 | Have you felt calm and peaceful? | Patient Health Questionnaire | Felt calm and peaceful |
| 230 | Did you have a lot of energy? | Patient Health Questionnaire | Have energy |
| 231 | Have you felt downhearted and blue? | Patient Health Questionnaire | Felt downhearted and blue |
| 232 | Did you feel worn out? | Patient Health Questionnaire | Feel worn out |
| 233 | Have you been a happy person? | Patient Health Questionnaire | Been happy |
| 234 | Did you feel tired? | Patient Health Questionnaire | Feel tired |
| 235 | During the past 4 weeks, how much of the time has your health limited your social activities (such as visiting with friends or close relatives)? | Health-related quality of life | Health limited social activities |
| 236 | Now please tell me the answer that best describes how true or false each of the following statements is for you.  I seem to get sick a little easier than other people. | Health-related quality of life | Get sick easier than others |
| 237 | I am as healthy as anybody I know. | Health-related quality of life | As healthy as anybody |
| 238 | I expect my health to get worse. | Health-related quality of life | Expect health to get worse |
| 239 | My health is excellent | Health-related quality of life | Excellent health |
| 240 | Comments for self-rated health | Health-related quality of life | Comments for self-rated health |
| 241 | Thinking about stress in your day-to-day life, what would you say is the most important thing contributing to feelings of stress you may have? | Stress | Largest daily life stress |
| 242 | Stress of everyday life - Other | Stress | Everyday stress |
| 243 | How much do you agree with the following statements:  When faced with this source of stress, I have the personal ability to deal with the situation and overcome it. | Stress | Personal abilities |
| 244 | When faced with this source of stress, I can count on people that I know to help me deal with the situation and overcome it. | Stress | Social network |
| 245 | In dealing with this source of stress, how would you rate the importance of your own ability to cope compared with the support of the people you know? | Stress | Relative share personal capacity and network |
| 246 | In general, how would you rate your ability to handle the day-to-day demands in your life, for example, handling work, family and volunteer responsibilities? | Stress | Ability to handle daily demands |
| 247 | In general, how would you rate your ability to handle unexpected and difficult problems? | Stress | Ability to handle unexpected problems |
| 248 | Thinking about the ways you deal with the stress  you identified, please tell me how often you do each of the following | Stress Management | Try to solve problems |
| 249 | To deal with stress, how often do you talk to others? | Stress Management | Speaks to others |
| 250 | When dealing with stress, how often do you avoid being with people? | Stress Management | Avoid company |
| 251 | How often do you sleep more than usual to deal with stress? | Stress Management | Sleeping more |
| 252 | When dealing with stress, how often do you try to feel better by eating more, or less, than usual? | Stress Management | Changing eating habits |
| 253 | When dealing with stress, how often do you try to feel better by smoking more cigarettes than usual? | Stress Management | More smoke |
| 254 | When dealing with stress, how often do you try to feel better by drinking alcohol? | Stress Management | Alcohol consumption |
| 255 | When dealing with stress, how often do you try to feel better by using drugs or medication? | Stress Management | Drugs/medication usage |
| 256 | How often do you jog or do other exercise to deal with stress? | Stress Management | Physical exercise |
| 257 | How often do you pray or seek spiritual help to deal with stress? | Stress Management | Spiritual help |
| 258 | To deal with stress, how often do you try to relax by doing something enjoyable? | Stress Management | Do something enjoyable |
| 259 | To deal with stress, how often do you try to look on the bright side of things? | Stress Management | Be positive |
| 260 | How often do you blame yourself? | Stress Management | Blames |
| 261 | To deal with stress, how often do you wish the situation would go away or somehow be over? | Stress Management | Magical thinking |
| 262 | During the last 12 months, did you lose anything that had sentimental value? | Life’s events | Lose sentimental value |
| 263 | Losing something sentimental value – how do you find this? | Life’s events | Stress assessment for losing sentimental value |
| 264 | During the last 12 months, did someone close to you die? | Life’s events | Deceased close |
| 265 | Deceased loved-  how do you find this? | Life’s events | Stress assessment for deceased loved |
| 266 | During the last 12 months, was someone you care about seriously ill? | Life’s events | Some one close seriously ill |
| 267 | Someone seriously ill - Did you find this stressful? | Life’s events | Stress assessment for gravely ill |
| 268 | During the last 12 months, were you seriously ill? | Life’s events | Seriously ill |
| 269 | You were seriously ill - Did you find this stressful? | Life’s events | Stress assessment for seriously ill |
| 270 | During the last 12 months, did you move? | Life’s events | Moved |
| 271 | I moved - Did you find this stressful? | Life’s events | Stress assessment for moving |
| 272 | During the last 12 months, did you have any problems with your friends or your neighbours? | Life’s events | Problems with friends or neighbors |
| 273 | Problems with friends or neighbors - Did you find this stressful? | Life’s events | Stress assessment for such problems |
| 274 | During the last 12 months, were you separated or divorced? | Life’s events | Separation or divorce |
| 275 | Separation or divorce - Did you find this stressful? | Life’s events | Stress assessment for separation or divorce |
| 276 | During the last 12 months, did you go through a break-up (other than separation or divorce)? | Life’s events | Breakup |
| 277 | Breakup - Did you find this: | Life’s events | Stress assessment for breakup |
| 278 | During the last 12 months, did you have to cut your ties with your children? | Life’s events | Broke ties with children |
| 279 | Breaking ties with his children - Did you find this stressful? | Life’s events | Stress assessment for such break |
| 280 | During the last 12 months, were you fired or dismissed from your job, or did you have problems at work? | Life’s events | Problems at work |
| 281 | Fired or troubles with employer - Did you find this stressful? | Life’s events | Stress assessment for problems at work |
| 282 | During the last 12 months, were you unemployed for a month or more? | Life’s events | Unemployed for a month |
| 283 | Unemployed for a month - Did you find this stressful? | Life’s events | Stress assessment for unemployment |
| 284 | During the last 12 months, did you declare personal bankruptcy? | Life’s events | Bankruptcy |
| 285 | Personal Bankruptcy - Did you find this stressful? | Life’s events | Stress assessment for bankruptcy |
| 286 | During the last 12 months, has your income decrease a lot? | Life’s events | Income drop |
| 287 | Significant drop in income - Did you find this stressful? | Life’s events | Stress assessment for income drop |
| 288 | During the last 12 months, did you get into serious debt? | Life’s events | Significant debt |
| 289 | Significant debt - Did you find this stressful? | Life’s events | Stress assessment for significant debt |
| 290 | During the last 12 months, were you physically abused (beaten, attacked)? | Life’s events | Physically abused |
| 291 | Physically abused - Did you find this stressful? | Life’s events | Stress assessment for physical abuse |
| 292 | During the last 12 months, were you robbed? | Life’s events | Robbed |
| 293 | Robbed - Did you find this stressful? | Life’s events | Stress assessment for robbery |
| 294 | During the last 12 months, were you sexually assaulted (raped)? | Life’s events | Rape victim |
| 295 | Raped - Did you find this stressful? | Life’s events | Stress assessment for being rapped |
| 296 | During the last 12 months, did you have any problems with the place where you were living (for example, it was condemned, there was a fire, or a problem with heating, electricity or plumbing)? | Life’s events | Problems with living places |
| 297 | Plumbing problems, heating, fire - Did you find this stressful? | Life’s events | Stress assessment for such problems |
| 298 | During the last 12 months, were you evicted from the place where you were living? | Life’s events | Eviction |
| 299 | Ousted from its housing- Did you find this stressful? | Life’s events | Stress assessment for eviction |
| 300 | During the last 12 months, did you stop receiving welfare or unemployment? | Life’s events | Termination benefits |
| 301 | Termination Benefits - Did you find this stressful? | Life’s events | Stress assessment for termination benefits |
| 302 | During the last 12 months, were your ties to your family broken off? | Life’s events | Break family ties |
| 303 | Breaking ties with the family - Did you find this stressful? | Life’s events | Stress assessment for the such break |
| 304 | In the last 12 months, did you have any other problems that caused stress | Life’s events | Another difficulty |
| 305 | Can you describe the situation? | Life’s events | Describe another difficulty |
| 306 | Another problem - Did you find this stressful? | Life’s events | Stress assessment for difficulty |
| 307 | In general, how would you rate your ability to cope with the problems you’ve mentioned and the stress that they cause? | Life’s events | Coping ability |
| 308 | How much do you agree with the following statements:  When faced with this (these) event(s), I have the personal ability to deal with the situation and overcome it. | Life’s events | Personal abilities |
| 309 | When faced with this (these) source(s) of stress, I can count on people that I know to help me deal with the situation and overcome it | Life’s events | Social network |
| 310 | In dealing with this (these) source(s) of stress, how would you rate the importance of your own ability to cope compared with the support of the people you know?  Choose the letter from A to K that best describes how much depends on your own ability as compared with the support of the people you know. | Life’s events | Relative share personal capacity and network |
| 311 | Comments of the life’s events and stress management | Life’s events | Comments |
| 312 | In general, how important are religious or spiritual beliefs in your daily life? | Spirituality | Importance spirituality |
| 313 | To what extent does your spiritual values give you the strength to face everyday difficulties? | Spirituality | Spirituality gives strength |
| 314 | Comments for spirituality | Spirituality | Comments for spirituality |
| 315 | In the last 12 months, did you have had a drink of beer, wine, liquor or any other alcoholic beverage? | Alcohol consumption | Alcohol consumption |
| 316 | In the last 12 months, how often did you drink alcoholic beverages? | Alcohol consumption | Alcohol consumption frequency |
| 317 | Over the past 12 months, how often have you had 5 or more drinks of alcohol on one occasion? | Alcohol consumption | 5+ drinks |
| 318 | Have you ever taken a drink of alcohol? | Alcohol consumption | Had a drink |
| 319 | Have you ever taken at least 12 drinks per year? | Alcohol consumption | Had 12+ drinks per year |
| 320 | On days when you drank in the last 12 months, about how many drinks did you usually take a day? | Alcohol consumption | Number of drinks per day |
| 321 | Do you ever have a year in which you drank more than you have done in the last 12 months? | Alcohol consumption | A year drink more |
| 322 | Think of the years of your life during which you drank the most. During these years, how often have you usually had at least one drink of alcohol? | Alcohol consumption | How often at least one drink |
| 323 | On days when you drank, during these years, about how many drinks do you usually take a day? | Alcohol consumption | Number of drinks per day |
| 324 | First, is there ever a time in your life when your drinking or your excess alcohol (hangover) often affected your work or responsibilities at school, at work or at the House? | Alcohol consumption | Drinking affected work/responsibilities |
| 325 | Is there ever a time in your life when your drinking is causing arguments or other serious or repeated problems with your family, friends, neighbors or colleagues? | Alcohol consumption | Drinking caused augments/problems |
| 326 | Did you continue to drink even though it caused problems with these people? | Alcohol consumption | Drink ignoring problems |
| 327 | Are there any moments in your life when you were often under the influence of alcohol in situations where you could be injured (e), for example, by bicycle, driving a car or operating a machine? | Alcohol consumption | Get hurt due to drinking |
| 328 | Have you ever been arrested (e) by the police for driving while intoxicated or drunk? | Alcohol consumption | Get arrested due to drunk driving |
| 329 | How many times have you been arrested (e) by the police because of your drinking? | Alcohol consumption | Nbr arrest due to drunk driving |
| 330 | When was the last time you had drinking because of alcohol? | Alcohol consumption | How recently the problems due to drinking |
| 331 | Age of the last time having the problem due to drinking | Alcohol consumption | Age of the last time having the problem due to drinking |
| 332 | At one point in your life, have you ever felt such a strong desire to drink that you could not stop you drinking or has you been hard to think of anything else? | Alcohol consumption | A strong desire to drink |
| 333 | Have you ever needed to consume greater amounts of alcohol to feel an effect, or have you already found that the amount of alcohol you consume before have you had more effect? | Alcohol consumption | Drink more to get effects |
| 334 | Have you ever had moments where when you stop, diminish your drinking or do not take alcohol, you will experience some withdrawal symptoms such as fatigue, headache, diarrhea, of shakes or emotional problems? | Alcohol consumption | Withdrawal symptoms |
| 335 | Have you had times when you took a drink to avoid having such problems? | Alcohol consumption | Drink to avoid withdrawal symptoms |
| 336 | Have you had times when you started drinking even though you promised yourself (e) that you would not do, or times when you used a lot more than you had planned? | Alcohol consumption | Still drink even though promise not to drink |
| 337 | Have you had times when you drank more frequently or for more days in a row than you had planned? | Alcohol consumption | Drank more |
| 338 | Have you had times when you started drinking and you became (e) drunk (e) when you do not want to? | Alcohol consumption | Drinking without wanting |
| 339 | Have you had times when you tried to stop drinking or reduce your drinking and found that you could not do? | Alcohol consumption | Failed to stop drinking |
| 340 | Have you ever experienced periods of several days or more when you spent so much time drinking alcohol or recovering from the effects that you had little time to do something else? | Alcohol consumption | Had periods spent time drinking |
| 341 | Have you ever had a time when you gave up or greatly reduced important activities because of your drinking, such as sports, work or visit friends and family? | Alcohol consumption | Cut down activities due to drinking |
| 342 | Have you ever continue to drink alcohol while knowing that you had a serious physical or emotional problem that might be caused or  worse by your drinking? | Alcohol consumption | Drinking when had drinking problems |
| 343 | Have you ever had three or more of these problems in the same period of 12 months? | Alcohol consumption | 3+ problems |
| 344 | When was the last time you had problems? | Alcohol consumption | How recently having problems |
| 345 | How old were you the last time you had any of these problems? | Alcohol consumption | Age of the last time having problems |
| 346 | Number of times ever made a serious attempt to quit drinking | Alcohol consumption | Number of times made attempts to quit drinking |
| 347 | Over the past 12 months, how much she has your drinking interfere with your home responsibilities, like cleaning, shopping and taking care of the house or apartment? | Alcohol consumption | Drinking affected home responsibilities |
| 348 | How does your alcohol interfere with your ability to go to school? | Alcohol consumption | Drinking affected school attendance |
| 349 | How does your alcohol interfere with your ability to work at a job? | Alcohol consumption | Drinking affected work ability |
| 350 | Drinking interferes with ability to form, maintain close relationships with other | Alcohol consumption | Drinking affected close relationships |
| 351 | How does your alcohol interfere with your social life? | Alcohol consumption | Drinking affected social life |
| 352 | Over the past 12 months, about how many days out of 365 were you totally unable to work or carry out your normal activities because of your drinking? | Alcohol consumption | Number of days unable for work/activities |
| 353 | During your life, have you ever seen, in person or by telephone, a doctor or other professional about your drinking? (By other professional, we mean psychologists, psychiatrists, social workers,  counselors, spiritual advisors, homeopaths, acupuncturists, support group or other health professionals.) | Alcohol consumption | Sought professional help |
| 354 | In the last 12 months, have you received treatment from professionals for your drinking? | Alcohol consumption | Professional treatment for drinking |
| 355 | During your life, have you ever been hospitalized (e) for a night or more for your drinking? | Alcohol consumption | Hospitalized due to drinking |
| 356 | Comments for drinking behavior | Alcohol consumption | Comments for drinking behavior |
| 357 | Have you ever taken a tranquilizer or sedative for nonmedical purposes? | Drug use | Used nonmedical sedative or tranquilizer |
| 358 | Have you ever taken a sedative or tranquilizer that a doctor has prescribed? | Drug use | Used prescribed sedative or tranquilizer use |
| 359 | Did you feel that you could not stop taking the sedative or tranquilizer that was prescribed for you? | Drug use | Can’t quit prescribed sedative or tranquilizer |
| 360 | Have you taken sedative or tranquilizer in the last 12 months? | Drug use | Used predative or tranquilizer past year |
| 361 | How often (did you take a sedative or tranquilizer in the past 12 months)? | Drug use | How often used sedative or tranquilizer |
| 362 | Have you ever taken a stimulant for non-medical purposes? | Drug use | Used nonmedical stimulant |
| 363 | Have you ever taken a stimulant that was prescribed by a doctor? | Drug use | Used Prescribed stimulant |
| 364 | Did you think you could not stop taking the stimulant that was prescribed for you? | Drug use | Can’t quit prescribed stimulant |
| 365 | Have you taken a stimulant in the last 12 months? | Drug use | Used stimulant past year |
| 366 | How often (did you take a stimulant during the past 12 months)? | Drug use | How often used stimulant |
| 367 | Have you ever taken a painkiller for non-medical purposes? | Drug use | Used nonmedical pain killer |
| 368 | Have you ever taken a painkiller that a doctor has prescribed? | Drug use | Used prescribed pain killer |
| 369 | Have you thought you could not stop taking the pain medication that you were prescribed? | Drug use | Can’t quit prescribed pain killer |
| 370 | Have you taken in the last 12 months? | Drug use | Used pain killer past year |
| 371 | How often (did you take a painkiller in the last 12 months)? | Drug use | How often used pain killer |
| 372 | Have you ever used or tried marijuana or hashish? | Drug use | Used marijuana or hashish |
| 373 | Have you taken in the last 12 months? | Drug use | Used marijuana or hashish past year |
| 374 | How often (did you use marijuana or hashish in the past 12 months)? | Drug use | How often used marijuana or hashish |
| 375 | Have you ever experienced a time when you used marijuana or hashish more often than you did in the last 12 months? | Drug use | Used marijuana or hashish more |
| 376 | Thinking about the year you used marijuana or hashish often, how often have you taken? | Drug use | How often used marijuana or hashish most |
| 377 | During your life, how many times have you used marijuana or hashish? | Drug use | Number of times used marijuana or hashish |
| 378 | Have you used marijuana or hashish over 50 times in your life? | Drug use | LT-Used marijuana or hashish 50+ times |
| 379 | Have you ever used or tried cocaine in any form, including powder, crack, crack cocaine ("free base"), coca leaves or paste? | Drug use | Used cocaine |
| 380 | Have you taken cocaine in the last 12 months? | Drug use | Used cocaine past year |
| 381 | How often (did you use cocaine over the past 12 months)? | Drug use | How often used cocaine |
| 382 | Have you ever used or tried club drugs such as ecstasy, ketamine and MDMA? | Drug use | Used club drugs |
| 383 | Have you taken club drugs in the last 12 months? | Drug use | Used club drugs past year |
| 384 | How often (did you take club drugs in the last 12 months)? | Drug use | How often used club drugs |
| 385 | Have you ever used or tried hallucinogens, including LSD, mescaline, PCP, angel dust of, or magic mushrooms peyote? | Drug use | Used hallucinogens |
| 386 | Have you taken hallucinogens in the last 12 months? | Drug use | Used hallucinogens past year |
| 387 | How often (did you take hallucinogens in the past 12 months)? | Drug use | How often used hallucinogens |
| 388 | Have you ever used or tried heroin or opium? | Drug use | Used heroin or opium |
| 389 | Have you taken heroin or opium in the last 12 months? | Drug use | Used heroin or opium past year |
| 390 | How often (did you use heroin or opium in the past 12 months)? | Drug use | How often used heroin or opium |
| 391 | Have you ever taken inhalants or solvents such as nitrous oxide, glue, paint or gasoline? | Drug use | Used inhalants or solvents |
| 392 | Have you taken inhalants or solvents in the last 12 months? | Drug use | Used inhalants past year |
| 393 | How often (did you inhalants or solvents during the past 12 months)? | Drug use | How often used inhalants or solvents |
| 394 | Have you taken other illegal drugs? | Drug use | Used other illegal drugs |
| 395 | Specify other illegal drugs | Drug use | Specify other illegal drugs |
| 396 | Ever used any illegal drug in the past 12 months? | Drug use | Used other illegal drug past year |
| 397 | How often used the illegal drug in the past 12 months? | Drug use | How often used other illegal drugs |
| 398 | Have you ever been a time in your life where your marijuana or hashish often conflicted with your work or responsibilities at school, at work or at home? | Drug use | Marijuana or hashish affected work/responsibilities |
| 399 | Have you ever been a time in your life where your marijuana or hashish was originally disputes or other serious or repeated problems with your family, friends or colleagues? | Drug use | Marijuana or hashish caused problem |
| 400 | Did you continue to take marijuana or hashish even though it caused problems with these people? | Drug use | Using marijuana/hashish ignoring problems |
| 401 | Are there has been times in your life when you  were often under the influence of marijuana or hashish in situations where you might have been injured (e), for example, by bicycle, driving a car or operating a machine? | Drug use | Get hurt due to using marijuana/hashish |
| 402 | Have you been stopped (e) through more than once for driving under the influence of marijuana or hashish police or because of your behavior under the influence of marijuana or hashish? | Drug use | Get arrested due to driving while using marijuana/hashish |
| 403 | How recently had problem because of using marijuana or hashish? | Drug use | How recently the problems due to marijuana or hashish |
| 404 | Age of last time having problem because of marijuana? | Drug use | Age of the last time having the problem due to marijuana |
| 405 | Had a strong desire to use marijuana or hashish that you couldn’t stop using? | Drug use | A strong desire to use marijuana or hashish |
| 406 | Have you ever needed to consume greater amounts of marijuana or hashish to feel an effect, or have you ever noticed that you no longer feel the usual effects with the amounts that you used to use? | Drug use | Used marijuana or hashish more to get effects |
| 407 | Have you had times when you stopped or decreased your consumption or does not take marijuana or hashish and you felt withdrawal symptoms? | Drug use | Withdrawal symptoms |
| 408 | Have you ever been times when you have used marijuana or hashish to avoid having such problems? | Drug use | Used marijuana or hashish to avoid withdrawal symptoms |
| 409 | Have you ever been times when you have used marijuana or hashish even if you had planned not to do so, or times when you've consumed a lot more than you had planned? | Drug use | Used marijuana or hashish though plan not |
| 410 | Have you ever been times when you have used marijuana or hashish more frequently or for more days in a row than you had planned? | Drug use | Used marijuana or hashish more |
| 411 | Are there any times when you tried to stop or reduce your use of marijuana or hashish and you have found that you could not do? | Drug use | Failed to stop using marijuana or hashish |
| 412 | Have you ever experienced periods of several days or more when you spent so much time consuming or recover from the effects of marijuana or hashish you had little time to do something else? | Drug use | Spent time using marijuana or hashish |
| 413 | Have you ever had a time when you gave up or greatly reduced important activities because of your use of marijuana or hashish - like sports, work or visit friends and family? | Drug use | Cut down activities due to using marijuana or hashish |
| 414 | Have you continued to use marijuana or hashish knowing that you had a serious physical or emotional problem that might be caused or made worse by your use of marijuana or hashish? | Drug use | Using marijuana or hashish when had problems |
| 415 | Have you ever had three or more of these problems during the same 12 month period when you consume marijuana or hashish? | Drug use | Had 3+ problems when using marijuana or hashish |
| 416 | How recently had problems because of using marijuana or hashish | Drug use | How recently the problems due to marijuana or hashish |
| 417 | How old were you the last time you had any of these problems because of your marijuana or hashish? | Drug use | Age of the last time having the problem due to marijuana |
| 418 | Using of drug frequently interfered with you work or responsibility? | Drug use | Drug affected work or responsibilities |
| 419 | Using of drug causing arguments or problems with others? | Drug use | Drug caused problem |
| 420 | Continued to use the drug even though it caused problems with others? | Drug use | Using drug ignoring problems |
| 421 | Often under the influence of drug in situation where could get hurt? | Drug use | Get hurt due to using drug |
| 422 | Arrested/stopped by police >once due to driving under the influence of the drug? | Drug use | Get arrested due to driving while using drug |
| 423 | How recently had problem because of using the drug? | Drug use | How recently the problems due to drug |
| 424 | Age of last time having problem due to the drug use? | Drug use | Age of the last time having the problem due to drug |
| 425 | Had such a strong desire to use the drug that could not stop? | Drug use | A strong desire to use drug |
| 426 | Needed larger amounts of the drug to get an effect that used to have? | Drug use | Used drug more to get effects |
| 427 | Experienced withdrawal symptoms after stopping using the drug? | Drug use | Withdrawal symptoms |
| 428 | Used the drug to keep from withdrawal symptoms? | Drug use | Used drug to avoid withdrawal symptoms |
| 429 | Used the drug even though planned not to use a lot more than intended? | Drug use | Used drug though plan not |
| 430 | Use the drug more frequently? | Drug use | Used drug more |
| 431 | Tried to cut down on use of the drug but not able to do so? | Drug use | Failed to stop using drug |
| 432 | Spent so much time using or recovering from the effects of using the drug? | Drug use | Spent time on using drug |
| 433 | Gave up or greatly reduced important activities because of using the drug? | Drug use | Cut down activities due to using drug |
| 434 | Continued to use the drug knowing having a serious physical/emotional problem? | Drug use | Using when had drug-related problems |
| 435 | Had 3+ these problems in the same 12 month period during the time using the drug? | Drug use | 3+ problems |
| 436 | When was the last time you had problems because of using the drug? | Drug use | How recently the problems due to drug |
| 437 | Age of last time having any of the problems due to using the drug | Drug use | Age of the last time having the problem due to drug |
| 438 | Using drug interfere with home responsibilities in the past 12 months | Drug use | Drug affected home responsibilities |
| 439 | How your consumption does it interfere with your ability to attend school? | Drug use | Drug affected school attendance |
| 440 | How your consumption does it interfere with your ability to work at a job? | Drug use | Drug affected affects work ability |
| 441 | Using drug interfere with ability to form and maintain relationship with others | Drug use | Drug affected close relationships |
| 442 | How does your drug use it interfere with your social life? | Drug use | Drug affected affects social |
| 443 | Number of days in the past 12 months unable to work/carry out normal activities | Drug use | Number of days unable for work/activities |
| 444 | Ever sought professional help about drug use | Drug use | Sought professional help |
| 445 | In the last 12 months, have you received treatment from professionals for your drug use? | Drug use | Professional treatment for using drug |
| 446 | During your life, have you ever been hospitalized (e) for a night or more for your drug use? | Drug use | Hospitalized for using drug |
| 447 | Additional comments for drug use? | Drug use | Additional comments for drug use? |
| 448 | Did somebody in your close family have ever suffered from a serious mental illness, emotional or psychological problems or problems related to alcohol or drugs? | Familial History of Mental Illness | Family history of mental illness |
| 449 | With whom did you spent most of your childhood? | Familial History of Mental Illness | Live with biological parents |
| 450 | With whom did you spent most of your childhood? Other relatives | Familial History of Mental Illness | Live with other relatives |
| 451 | Has anyone in your immediate family ever had a serious mental illness, emotional problem or nervous breakdown? | Familial History of Mental Illness | Family history |
| 452 | Has anyone in your immediate family ever had a serious mental illness, emotional problem, or nervous breakdown? | Familial History of Mental Illness | Non-biological family history |
| 453 | Has anyone in your immediate family ever seen a psychiatrist, a psychologist, a social worker, or other health professional for a psychological or emotional problem? | Familial History of Mental Illness | Family mental problem consultation |
| 454 | Has anyone in your immediate family ever stayed overnight or longer in a hospital or treatment facility because of any mental or emotional problem? | Familial History of Mental Illness | Family inpatient mental health problem |
| 455 | Has anyone in your immediate family ever have a period in their life when they drank a lot? | Familial History of Mental Illness | Family drinking too much |
| 456 | Has anyone in your immediate family ever seen a psychiatrist, a psychologist, a social worker or other health professional for problems related to their alcohol consumption? | Familial History of Mental Illness | Family alcohol consultation |
| 457 | Has anyone in your immediate family ever had a period in their life when they used illegal drugs regularly? | Familial History of Mental Illness | Family illegal drugs |
| 458 | Has anyone in your immediate family ever seen a psychiatrist, a psychologist, a social worker, or other health professional for problems related to  there illegal drug use? | Familial History of Mental Illness | Family drug use consultation |
| 459 | Has anyone in your immediate family ever been in a detox center because of a problem with drugs or alcohol? | Familial History of Mental Illness | Family rehab center |
| 460 | Comments to family of mental illness | Familial History of Mental Illness | Comments |
| 461 | In the past 12 months, did you take any medication to help you with problems with your emotions, mental health or use of alcohol or drugs? | Medications for 12 months | Mental problems medications past 12-month |
| 462 | During those 2 days, how many different medications did you take for problems with your emotions, mental health, or use of alcohol or drugs? | Medications for past 2 days | Mental problems medications past 2 days |
| 463 | DIN available for medication no. 1 | Medications | DIN available for medication no. 1 |
| 464 | DIN for medication no. 1 | Medications | DIN for medication no. 1 |
| 465 | DIN for medication no. 1 | Medications | DIN for medication no. 1 |
| 466 | Exact name of medication no. 1 | Medications | Exact name of medication no. 1 |
| 467 | Exact name of medication no. 1 | Medications | Exact name of medication no. 1 |
| 468 | DIN available for medication no. 2 | Medications | DIN available for medication no. 2 |
| 469 | DIN for medication no. 2 | Medications | DIN for medication no. 2 |
| 470 | DIN for medication no. 2 | Medications | DIN for medication no. 2 |
| 471 | Exact name of medication no. 2 | Medications | Exact name of medication no. 2 |
| 472 | Exact name of medication no. 2 | Medications | Exact name of medication no. 2 |
| 473 | DIN available for medication no. 3 | Medications | DIN available for medication no. 3 |
| 474 | DIN for medication no. 3 | Medications | DIN for medication no. 3 |
| 475 | DIN for medication no. 3 | Medications | DIN for medication no. 3 |
| 476 | Exact name of medication no. 3 | Medications | Exact name of medication no. 3 |
| 477 | Exact name of medication no. 3 | Medications | Exact name of medication no. 3 |
| 478 | DIN available for medication no. 4 | Medications | DIN available for medication no. 4 |
| 479 | DIN for medication no. 4 | Medications | DIN for medication no. 4 |
| 480 | DIN for medication no. 4 | Medications | DIN for medication no. 4 |
| 481 | Exact name of medication no.4 | Medications | Exact name of medication no.4 |
| 482 | Exact name of medication no.4 | Medications | Exact name of medication no.4 |
| 483 | DIN available for medication no. 5 | Medications | DIN available for medication no. 5 |
| 484 | DIN for medication no. 5 | Medications | DIN for medication no. 5 |
| 485 | DIN for medication no. 5 | Medications | DIN for medication no. 5 |
| 486 | Exact name of medication no. 5 | Medications | Exact name of medication no. 5 |
| 487 | Exact name of medication no. 5 | Medications | Exact name of medication no. 5 |
| 488 | DIN available for medication no. 6 | Medications | DIN available for medication no. 6 |
| 489 | DIN for medication no. 6 | Medications | DIN for medication no. 6 |
| 490 | DIN for medication no. 6 | Medications | DIN for medication no. 6 |
| 491 | Exact name of medication no.6 | Medications | Exact name of medication no.6 |
| 492 | Exact name of medication no.6 | Medications | Exact name of medication no.6 |
| 493 | DIN available for medication no. 7 | Medications | DIN available for medication no. 7 |
| 494 | DIN for medication no. 7 | Medications | DIN for medication no. 7 |
| 495 | DIN for medication no. 7 | Medications | DIN for medication no. 7 |
| 496 | Exact name of medication no. 7 | Medications | Exact name of medication no. 7 |
| 497 | Exact name of medication no. 7 | Medications | Exact name of medication no. 7 |
| 498 | DIN available for medication no. 8 | Medications | DIN available for medication no. 8 |
| 499 | DIN for medication no. 8 | Medications | DIN for medication no. 8 |
| 500 | DIN for medication no. 8 | Medications | DIN for medication no. 8 |
| 501 | Exact name of medication no. 8 | Medications | Exact name of medication no. 8 |
| 502 | Exact name of medication no. 8 | Medications | Exact name of medication no. 8 |
| 503 | DIN available for medication no. 9 | Medications | DIN available for medication no. 9 |
| 504 | DIN for medication no. 9 | Medications | DIN for medication no. 9 |
| 505 | DIN for medication no. 9 | Medications | DIN for medication no. 9 |
| 506 | Exact name of medication no. 9 | Medications | Exact name of medication no. 9 |
| 507 | Exact name of medication no. 9 | Medications | Exact name of medication no. 9 |
| 508 | Do you have insurance that covers all or part of the cost of your prescription medication? Include any private, government or employee-paid insurance plans? | Medications | Whether have insurance |
| 509 | In the past 12 months, have you used any of these health products? | Medications | Used other health products |
| 510 | Comments for medications | Medications | Comments for medications |
| 511 | Did you take sleeping pills in the past 12 months? | Use of psychotropic drugs | Sleeping pills |
| 512 | Did you take anxiolytics in the past 12 months? | Use of psychotropic drugs | Anxiolytics |
| 513 | Did you take mood stabilizers in the past 12 months? | Use of psychotropic drugs | Mood stabilizers |
| 514 | Did you take antidepressants in the past 12 months? | Use of psychotropic drugs | Antidepressants |
| 515 | Did you take antipsychotics in the past 12 months? | Use of psychotropic drugs | Antipsychotics |
| 516 | Did you take stimulants in the past 12 months? | Use of psychotropic drugs | Stimulants |
| 517 | Took medication for emotion mental health alcohol/drug use problems. What is the name of medication? | Use of psychotropic drugs | Medication name |
| 518 | Took medication for emotion mental health alcohol/drug use problems. What is the name of medication? | Use of psychotropic drugs | Medication name |
| 519 | Took medication for emotion mental health alcohol/drug use problems. What is the name of medication? | Use of psychotropic drugs | Medication name |

**Figure S1**. Pascal gene scores comparison between MDD probability and MDD diagnosis
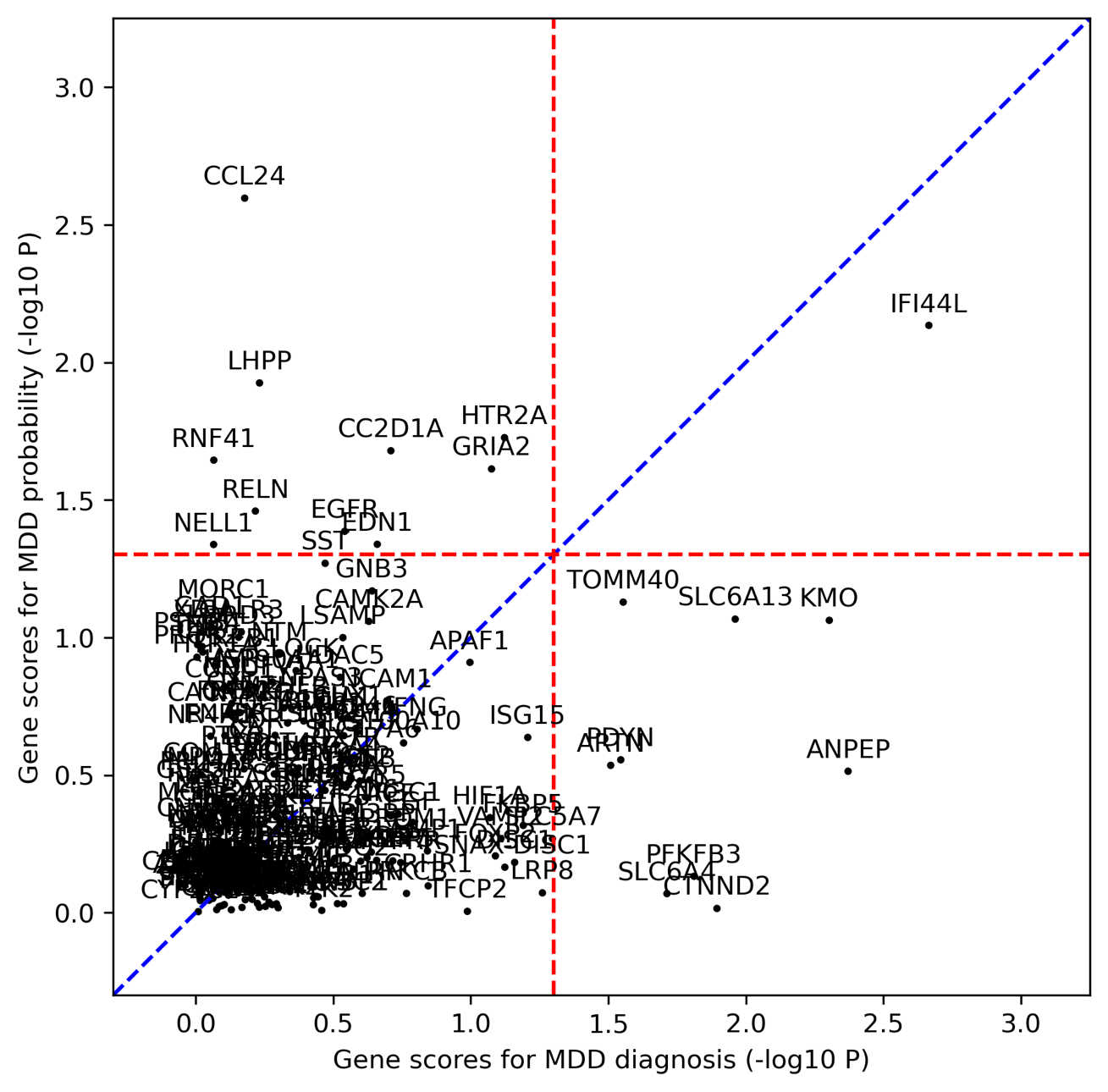

Supplement: Supplementary file 1 — Supplemental materials [file 41398_2022_2015_MOESM1_ESM.docx]
